# Load balance -aware dynamic cloud-edge-end collaborative offloading strategy

**DOI:** 10.1371/journal.pone.0296897

**Published:** 2024-01-12

**Authors:** Yueqi Fan

**Affiliations:** Shanxi Polytechnic College, Taiyuan, Shanxi, China; TU Wien: Technische Universitat Wien, AUSTRIA

## Abstract

Cloud-edge-end (CEE) computing is a hybrid computing paradigm that converges the principles of edge and cloud computing. In the design of CEE systems, a crucial challenge is to develop efficient offloading strategies to achieve the collaboration of edge and cloud offloading. Although CEE offloading problems have been widely studied under various backgrounds and methodologies, load balance, which is an indispensable scheme in CEE systems to ensure the full utilization of edge resources, is still a factor that has not yet been accounted for. To fill this research gap, we are devoted to developing a dynamic load balance -aware CEE offloading strategy. First, we propose a load evolution model to characterize the influences of offloading strategies on the system load dynamics and, on this basis, establish a latency model as a performance metric of different offloading strategies. Then, we formulate an optimal control model to seek the optimal offloading strategy that minimizes the latency. Second, we analyze the feasibility of typical optimal control numerical methods in solving our proposed model, and develop a numerical method based on the framework of genetic algorithm. Third, through a series of numerical experiments, we verify our proposed method. Results show that our method is effective.

## 1 Introduction

Due to the fast advancement of micro-computer technology, Internet-of-Things (IoT) devices have gained widespread adoption for data collection [1]. However, because of their limitations in energy and computation capability, IoT devices face great challenges in supporting resource-intensive applications. In this context, edge computing, a computation paradigm that enables IoT tasks to be processed at the edge of the Internet, has been suggested for effective computation offloading and has become a foundational technology in IoT architectures [2, 3].

Because of many objective factors such as construction costs, the power of edge servers is generally far inferior to that of cloud centers. In this context, though edge computing can process IoT tasks with low latency, the constrained capability of edge servers is still an Achilles heel when edge computing has to tackle immense amounts of data. To address this issue, a hybrid computation paradigm, known as cloud-edge-end (CEE) computing [4–6], has been proposed in recent years.

**Fig 1:**
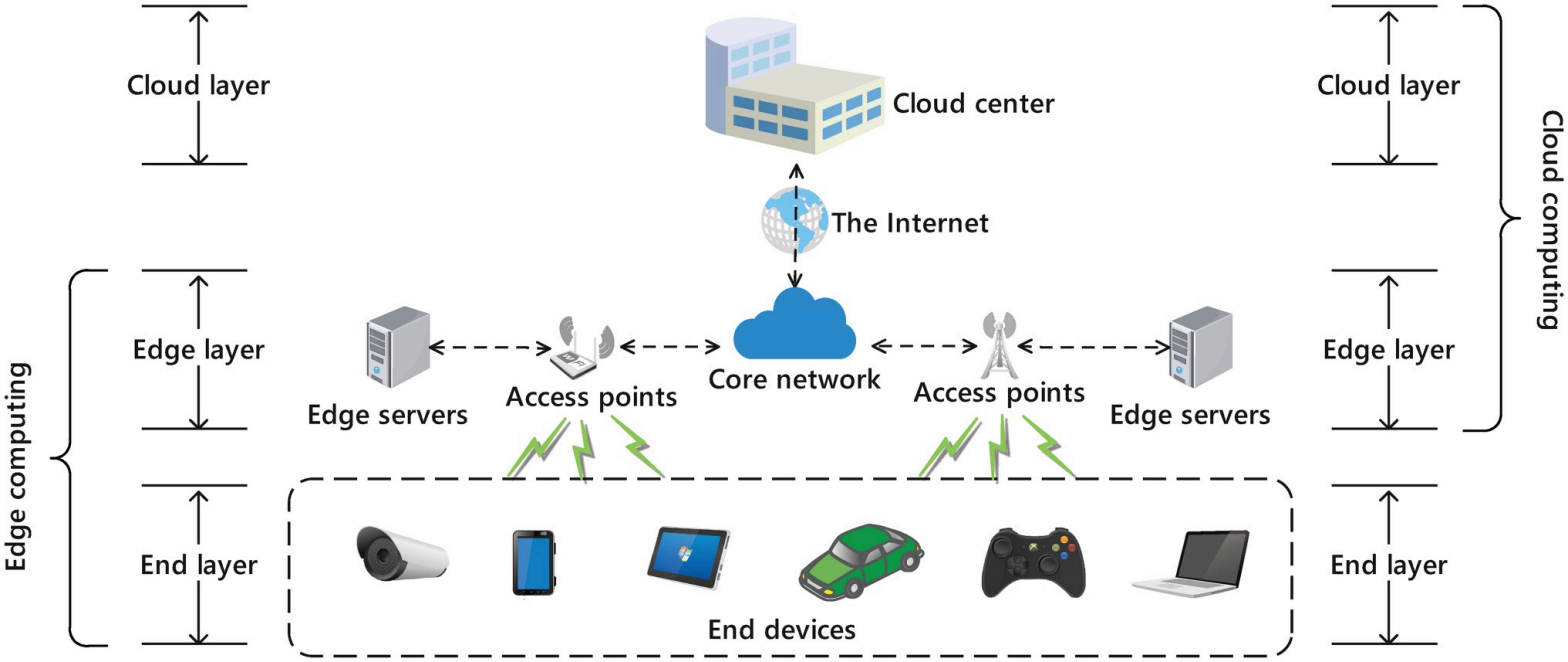
Diagram of common CEE systems.

**Fig 2:**
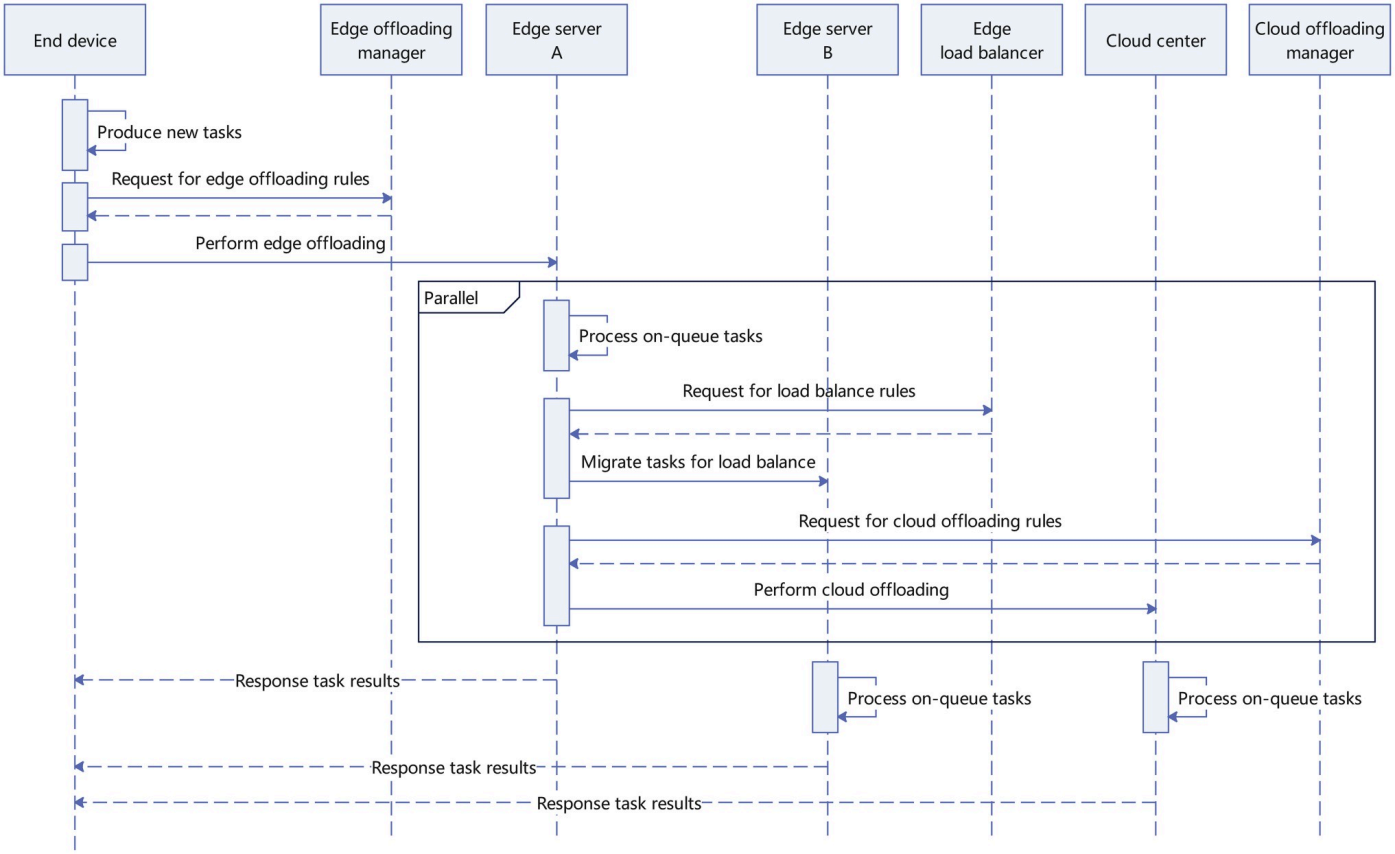
Sequence chart for workflow of CEE systems.

As illustrated in Fig 1, a CEE system is built on a hierarchical architecture and is generally composed of a resource-rich cloud center, numerous capability-constrained edge servers, and a huge number of end devices. From a bottom-up view, end devices are connected to edge servers through the core network, and edge servers are connected to the cloud center through the Internet. A typical workflow of the CEE system can be described as follows. First, computation tasks are constantly produced by end devices, and end devices will offload these tasks to edge servers through the core network under the control of an edge offloading scheme. Then, edge servers receive the arriving tasks and push them into a built-in queue. Next, in each edge server, a proportion of on-queue tasks are processed locally, while the remaining tasks will be either migrated to other edge servers under the control of a load balance scheme, or further offloaded to the cloud center for remote assistance under the control of a cloud offloading scheme. Finally, after being processed, tasks are responded to end devices from edge servers or the cloud center. A detail sequence chart describing the above steps is given in Fig 2.

From a macroscopic perspective, CEE computing converges the design principles of cloud computing and edge computing—end devices and edge servers together compose a typical edge computing system, while edge servers and the cloud center compose a cloud computing system. On one hand, compared with edge computing, CEE computing integrates sufficient resources from the cloud center, so that when edge servers are overloaded, a proportion of tasks on edge servers can be further offloaded to the cloud center for load reduction; on the other hand, compared with cloud computing, CEE computing provides low-latency services for end devices through numerous edge servers. Thus, CEE computing is a promising combination of edge and cloud computing [7, 8].

### 1.1 Research statement

Computation offloading, the manner in which resource-intensive tasks are migrated from a resource-constrained device to a resource-rich infrastructure, is a crucial design issue in CEE systems [8]. In this paper, we are devoted to developing efficient CEE offloading strategies that are aware of load balance. More specifically, we consider the following problem:

*Load balance -aware offloading (LBAO) problem: Consider a CEE system composed of a cloud center, multiple edge servers, and multiple end-device collections. Suppose the task producing rate of each end-device collection is known or predictable. Suppose load balance is available in the CEE system. Then, for a finite time horizon, how to collaboratively and dynamically determine the cloud offloading rates of edge servers and the many-to-many edge offloading proportions between end-device collections and edge servers, such that the total task latency is minimized?*

### 1.2 Contribution

To address the LBAO problem, our contributions are as follows.

- On mathematical modeling, we propose a load evolution model to characterize the influences of different CEE offloading strategies on a CEE system, establish a latency model as the performance criterion of CEE offloading strategies, and reduce the LBAO problem to an optimal control model.
- On problem solving, we analyze the feasibility of typical optimal control numerical methods in solving the LBAO problem. Then, based on the framework of genetic algorithm (GA) [9], we develop a numerical algorithm called the LBAO algorithm to solve the LBAO problem, and make a rough analysis of the time complexity of the LBAO algorithm.
- On simulations, we perform a series of numerical experiments to verify the proposed LBAO algorithm. First, we discuss the parameter setting of our experiments. Second, we investigate the optimal configuration of the LBAO algorithm. Third, by comparing the LBAO algorithm with other commonly used methods, we examine the performance of the LBAO algorithm. Finally, we give insight into the influence of load balance in CEE collaborative offloading.

The remainder of this paper is organized in the following manner. Section 2 reviews the related work. Section 3 presents the mathematical modeling of the LBAO problem. Section 4 discusses the solution to the LBAO problem. Section 5 shows numerical experiments. Finally, this paper is closed by Section 6.

## 2 Related work

In this section, we review the literature related to our work, and highlight the novelty of our work.

Typically, computation offloading is defined as the process in which computation tasks are migrated from a capability-constrained device to a resource-sufficient infrastructure to obtain remote assistance [10]. As computation offloading is an indispensable feature of edge computing, how to develop efficient edge offloading strategies is a crucial issue in the design of edge computing [11]. In the past years, edge offloading problems have been comprehensively investigated under various backgrounds and methodologies [12–15]. Below, some representative examples are sketched. In [12], offloading issues are studied under an edge-fog hierarchical network, and an incentive mechanism is designed to shift selfish users’ preference from the edge layer to the fog layer according to user delay tolerance. In [13], task-dependent offloading issues are investigated, and offloading strategies are jointly optimized based on a directed cyclic graph model that presents the dependence between tasks. Also, the literature [14] takes insight into the offloading problems for wireless powered edge networks and develops a reinforcement learning algorithm to attain effective binary offloading decisions. Besides, the research [15] proposes a lightweight mobility prediction and offloading framework by using a machine learning method, aiming to jointly handle computation offloading and mobility management issues in edge computing.

Although edge offloading problems have been extensively studied, the research on CEE offloading issues is still in an early stage. Different from the cases in edge computing, CEE offloading problems focus more on the collaboration of the edge offloading between end devices and edge servers and the remote cloud assistance between edge servers and cloud centers. In this context, the existing edge offloading strategies cannot be directly applied to solve CEE offloading problems, because the system architecture of edge computing rarely includes the remote connection between cloud centers and edge servers, which is exactly a core component of CEE systems. Thus, it is necessary to develop new schemes for CEE systems based on the existing edge offloading techniques. Towards this direction, a large number of CEE offloading strategies have been proposed [4, 6, 8, 16–23]. Below, some representative examples are sketched. In [4], efficient CEE offloading schemes are investigated in consideration of the joint optimization with resource allocation mechanisms. In [16, 18], hierarchical and horizontal CEE computing architectures are discussed, and CEE offloading problems are addressed through game theory. In [19], offloading issues are formulated as a multi-objective optimization problem and solved by using the Non-dominated Sorting Genetic Algorithm III (NSGA-III). In addition, the literature [23] focuses on the joint optimization of task offloading and service deployment for sequential tasks.

Unfortunately, as far as we know, CEE offloading strategies in the existing research are generally developed in consideration of transmission/propagation latency, energy consumption, task dropping rate, channel congestion, computation efficiency and financial payment, while load balance in CEE systems has rarely been accounted for. Load balance is an indispensable mechanism in CEE systems to guarantee a full utilization of resources of edge servers [24–26]. Typically, load balance is implemented based on a technique known as computation migration [27]; that is, when an edge server becomes overloaded due to the *flash crowd* caused by end devices (i.e., the network congestion which occurs when a huge number of end devices request the edge server simultaneously [28]), tasks on the queue of that edge server (or containers or virtual machines of the tasks) can be migrated to another edge server through a centralized management platform (e.g., software defined networking [29]) for load balance. Because there is such a non-negligible load balance mechanism in reality, the existing CEE offloading strategies may produce certain errors when estimating the load distribution of edge servers and may not be able to make the most efficient decisions. Therefore, it is necessary to propose a load balance -aware CEE offloading strategy.

To fill the above research gap, in this paper we are devoted to developing a load balance -aware CEE offloading strategy. The novelty of our work is sketched as follows. First, we propose a novel load evolution model to characterize the influences of different offloading strategies on the load balance of a CEE system. Second, we optimize a time-varying offloading strategy based on the system load distribution and overall latency. To our best knowledge, this is the first time to make these attempts.

## 3 Problem formulation

In this section, we present the mathematical formulation of the LBAO problem. First, we formalize the mathematical expression of CEE offloading strategies. Second, we propose a load evolution model to capture the effects of a CEE offloading strategy on a CEE system. Third, we establish a latency model to evaluate different CEE offloading strategies. Finally, we reduce the LBAO problem to a continuous-time optimal control model, with the CEE offloading strategy as the control variable, the latency model as the objective functional, and the load evolution model as a constraint.

### 3.1 Basic terms and notations

**Fig 3:**
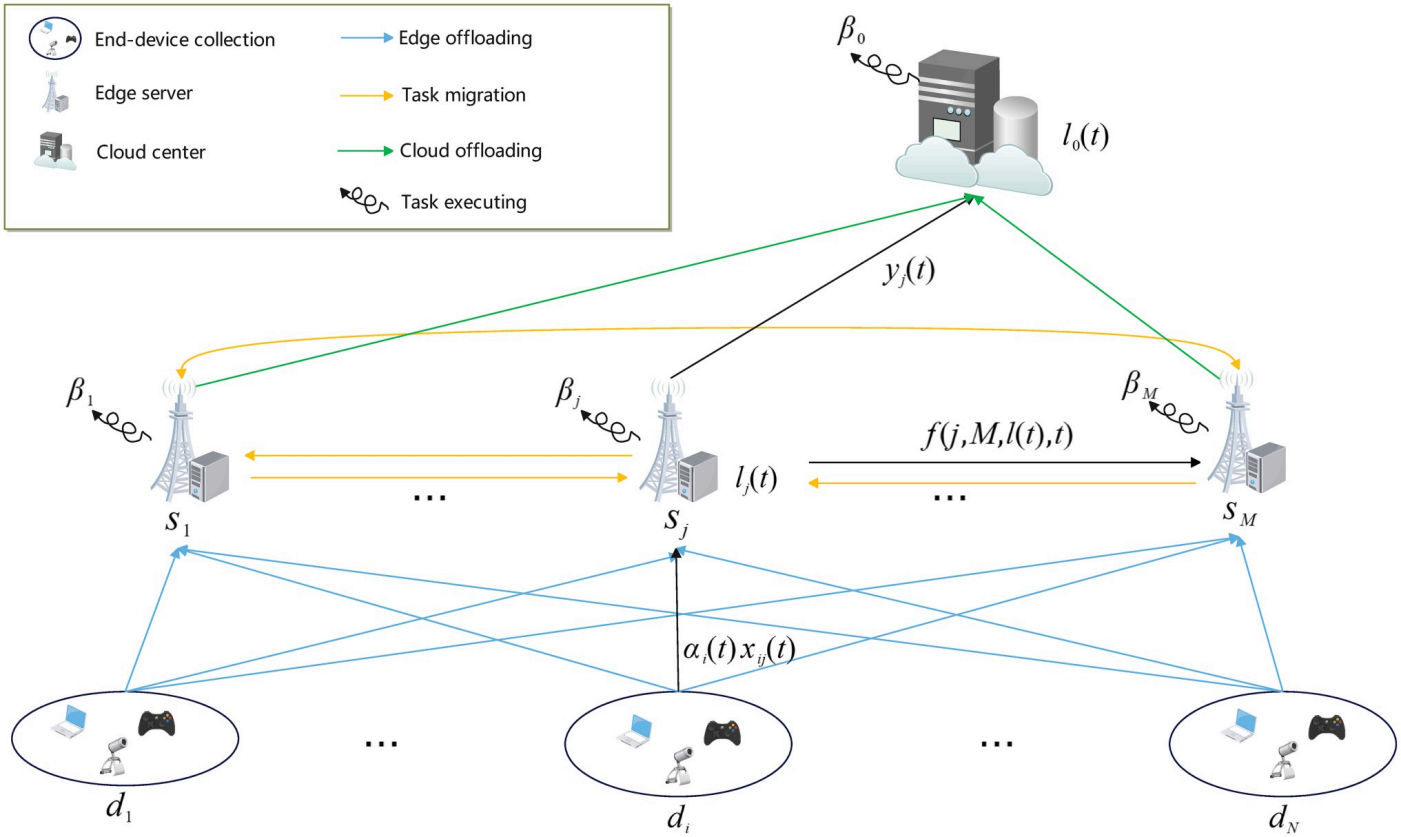
Illustration of notations.

Let us consider a CEE system composed of a remote cloud center, *M* edge servers, and *N* collections of end devices, as illustrated in Fig 3. Denote the set of the *M* edge servers by *S* = {*s*_1_, …, *s*_*M*_} and the set of the *N* end-device collections by *D* = {*d*_1_, …, *d*_*N*_}.

Consider a finite time horizon [0, *T*]. At any time *t* ∈ [0, *T*], denote *α*_*i*_(*t*) as the task producing rate of the end-device collection *d*_*i*_. Denote *x*_*ij*_(*t*) as the proportion of tasks offloaded from the end-device collection *d*_*i*_ to the edge server *s*_*j*_ at time *t*. By definition, $\sum_{j=1}^{M}x_{ij}\left(t\right)=1$ holds for all *i* and *t*. We refer to the function **x**(*t*) = [*x*_*ij*_(*t*)]_*N*×*M*_, 0 ≤ *t* ≤ *T*, as the *edge offloading strategy*. At time *t*, denote *l*_*j*_(*t*) as the *load* (i.e., the length of the task queue) of the edge server *s*_*j*_ and *l*_0_(*t*) as the load of the cloud center. Then, we refer to the function *l*(*t*) = (*l*_0_(*t*), *l*_1_(*t*), …, *l*_*M*_(*t*)), 0 ≤ *t* ≤ *T*, as the *load evolution trajectory*.

Once tasks have arrived at the queue of an edge server, they will be processed locally, migrated to other edge servers through a load balance scheme, or further offloaded to the cloud center for remote assistance. Suppose at any time, the task executing speed of a server is relatively stable. Thus, denote *β*_*j*_ as the average task processing rate of the edge server *s*_*j*_ and *β*_0_ as that of the cloud center. Also, denote the load balance scheme by *f* such that at time *t*, the edge server *s*_*i*_ will migrate its own tasks to the edge server *s*_*j*_ at the rate of *f*(*i*, *j*, *l*(*t*), *t*). Because it is no need for an edge server to migrate its own tasks to itself, let *f*(*i*, *i*, *l*(*t*), *t*) = 0 for all *i*. Moreover, denote *y*_*j*_(*t*) as the cloud offloading rate of the edge server *s*_*j*_ at time *t*. Then, we refer to the function *y*(*t*) = (*y*_1_(*t*), …, *y*_*M*_(*t*)), 0 ≤ *t* ≤ *T*, as the *cloud offloading strategy*. Denote *y*_max_ as the common maximum of cloud offloading rates.

Based on the above discussions, we combine together the edge offloading strategy **x** and cloud offloading strategy *y*, and refer to the function pair (**x**(*t*), *y*(*t*)), 0 ≤ *t* ≤ *T*, as the *CEE offloading strategy*. Clearly, the feasible set of CEE offloading strategies is

$$\begin{array}{c} \begin{array}{cc} \Omega = & \{\left(x,y\right):R\to R^{N\times M}\times R^{M}\;|\;0\leq x_{ij}\left(t\right)\leq 1,\;\sum_{m=1}^{M}x_{im}\left(t\right)=1,\\ & \;\;\;0\leq y_{j}\left(t\right)\leq y_{\text{max}},\;i=1,\cdots ,N,\;j=1,\cdots ,M,\;0\leq t\leq T\}. \end{array} \end{array}$$ (1)

**Fig 4:**
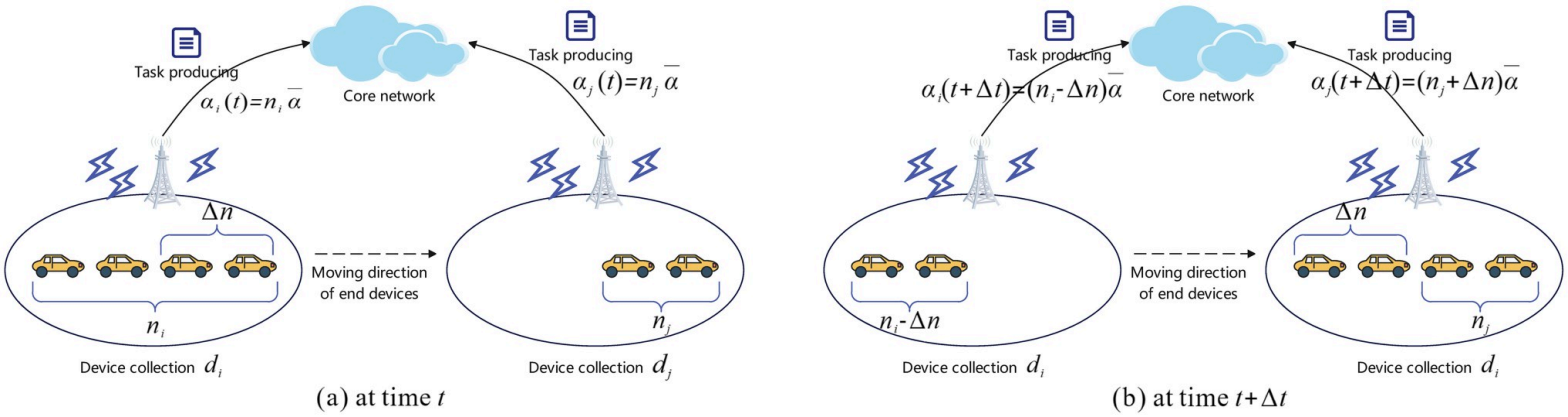
Illustration of user mobility.

**Remark 1** *In this paper, the user mobility in edge computing is presented by the time-varying task producing rates α*_*i*_(*t*). *To explain that, let us introduce a numerical example as follows. Denote* $\bar{\alpha }$ *as the average task producing rate of an end device at any time. Suppose there are n*_*i*_ *and n*_*j*_ *devices within the collections d*_*i*_ and *d*_*j*_ *at time t*, *respectively. Then, the task producing rates at time t are* $\alpha_{i}\left(t\right)=n_{i}\bar{\alpha }$ and $\alpha_{j}\left(t\right)=n_{j}\bar{\alpha }$. *If there are* Δ*n devices moving from d*_*i*_ to *d*_*j*_ *during the time interval* [*t*, *t* + Δ*t*], *then the task producing rates at time t* + Δ*t will be* $\alpha_{i}\left(t+\Delta t\right)=\left(n_{i}-\Delta n\right)\bar{\alpha }$ and $\alpha_{j}\left(t+\Delta t\right)=\left(n_{j}+\Delta n\right)\bar{\alpha }$. *A diagram illustrating the above scenario is shown in* Fig 4.

### 3.2 Load evolution model

Next, let us investigate the relationship between the CEE offloading strategy (**x**, *y*) and the load evolution trajectory *l*.

Suppose each server has a task queue of infinite length. In this paper, we refer to the term “*load dynamics*” as the trajectories of load changes on all servers in a CEE system. Below, we analyze the load dynamics of a CEE system.

According to the discussions in the previous subsection, the load of an edge server is determined by the following four aspects. Firstly, if the queue of the server is not empty, the load of the server will constantly decrease because tasks on the queue are being processed by the server. Secondly, the load of the server will increase because the server is receiving tasks from end devices. Thirdly, due to the load balance scheme, the server will migrate its own tasks to other servers or receive tasks from other servers. Finally, due to cloud offloading, the load will reduce if not empty.

Different from the case of edge servers, the load of the cloud center is only determined by the following two aspects. Firstly, if the queue of the cloud center is not empty, the load of the cloud center will decrease because of task executing. Secondly, because of cloud offloading, the load of the cloud center will increase.

Based on the above analysis, we derive a dynamical system in Theorem 1 to characterize the load dynamics of edge servers and the cloud center.

**Theorem 1** *Denote the initial load distribution of the CEE system by L*_0_. *Then, the load evolution trajectory l under the control of the CEE offloading strategy* (**x**, *y*) *satisfies the dynamical system* (2).

$$\begin{array}{c} \left\{ \begin{array}{cc} \dot{l_{0}}\left(t\right) & =\sum_{j=1}^{M}\Gamma \left(l_{j}\left(t\right)\right)y_{j}\left(t\right)-\Gamma \left(l_{0}\left(t\right)\right)\beta_{0},\;0\leq t\leq T,\\ \dot{l_{j}}\left(t\right) & =\sum_{i=1}^{N}x_{ij}\left(t\right)\alpha_{i}\left(t\right)-\Gamma \left(l_{j}\left(t\right)\right)\beta_{j}-\Gamma \left(l_{j}\left(t\right)\right)y_{j}\left(t\right)+\sum_{k=1}^{M}f\left(k,j,l\left(t\right),t\right)\\ & -\sum_{k=1}^{M}f\left(j,k,l\left(t\right),t\right),\;0\leq t\leq T,\;j=1,\cdots ,M,\\ l(0) & =L_{0}, \end{array}\right. \end{array}$$ (2)

*where* Γ(0) = 0, Γ(*x*) = 1, ∀*x* > 0.

**Proof 1** *Let* Δ*t be a variation of time. Consider the tiny time interval* [*t*, *t* + Δ*t*]. *In this time interval, the load of the cloud center will increase by* $\sum_{j=1}^{M}\Gamma \left(l_{j}\left(t\right)\right)y_{j}\left(t\right)\Delta t$ *because of cloud offloading and meanwhile decrease by* Γ(*l*_0_(*t*))*β*_0_Δ*t* *because of task processing. Thus, the load of the cloud center at time t* + Δ*t will be*

$$\begin{array}{c} l_{0}\left(t+\Delta t\right)=l_{0}\left(t\right)+\sum_{j=1}^{M}\Gamma \left(l_{j}\left(t\right)\right)y_{j}\left(t\right)\Delta t-\Gamma \left(l_{0}\left(t\right)\right)\beta_{0}\Delta t. \end{array}$$ (3)

*Because*

$$\begin{array}{c} {\dot{l}}_{0}\left(t\right)=\underset{\Delta t\to 0^{+}}{\text{lim}}\frac{l_{0}\left(t+\Delta t\right)-l_{0}\left(t\right)}{\Delta t},\;0\leq t\leq T, \end{array}$$ (4)

*the first equation in the dynamical system* (2) *is obtained by direction calculation*.

*Similarly, during the time interval* [*t*, *t* + Δ*t*], *the load of the edge server s*_*j*_ *will increase by* $\sum_{i=1}^{N}x_{ij}\left(t\right)\alpha_{i}\left(t\right)\Delta t$ *due to edge offloading, decrease by* Γ(*l*_*j*_(*t*))*β*_*j*_Δ*t* *and* Γ(*l*_*j*_(*t*))*y*_*j*_(*t*)Δ*t due to task consumption and cloud offloading, respectively, and change by* $[\sum_{k=1}^{M}f\left(k,j,l\left(t\right),t\right)-\sum_{k=1}^{M}f\left(j,k,l\left(t\right),t\right)]\Delta t$ *due to load balance. Because*

$$\begin{array}{c} \begin{array}{cc} l_{j}\left(t+\Delta t\right) & =l_{j}\left(t\right)+\sum_{i=1}^{N}x_{ij}\left(t\right)\alpha_{i}\left(t\right)\Delta t-\Gamma \left(l_{j}\left(t\right)\right)\beta_{j}\Delta t-\Gamma \left(l_{j}\left(t\right)\right)y_{j}\left(t\right)\Delta t\\ & +[\sum_{k=1}^{M}\;f\left(k,j,l\left(t\right),t\right)-\sum_{k=1}^{M}\;f\left(j,k,l\left(t\right),t\right)]\Delta t, \end{array} \end{array}$$ (5)

*and*

$$\begin{array}{c} {\dot{l}}_{j}\left(t\right)=\underset{\Delta t\to 0^{+}}{\text{lim}}\frac{l_{j}\left(t+\Delta t\right)-l_{j}\left(t\right)}{\Delta t},\;0\leq t\leq T, \end{array}$$ (6)

*the remaining equations in the dynamical system* (2) *are obtained from direct calculation. The proof is complete*.

The dynamical system (2) reveals the relationship between the CEE offloading strategy (**x**, *y*) and the load evolution trajectory *l*, with which we can predict the load dynamics of the CEE system under an arbitrary CEE offloading strategy. Thus, we refer to this dynamical system as the *load evolution model*.

**Fig 5:**
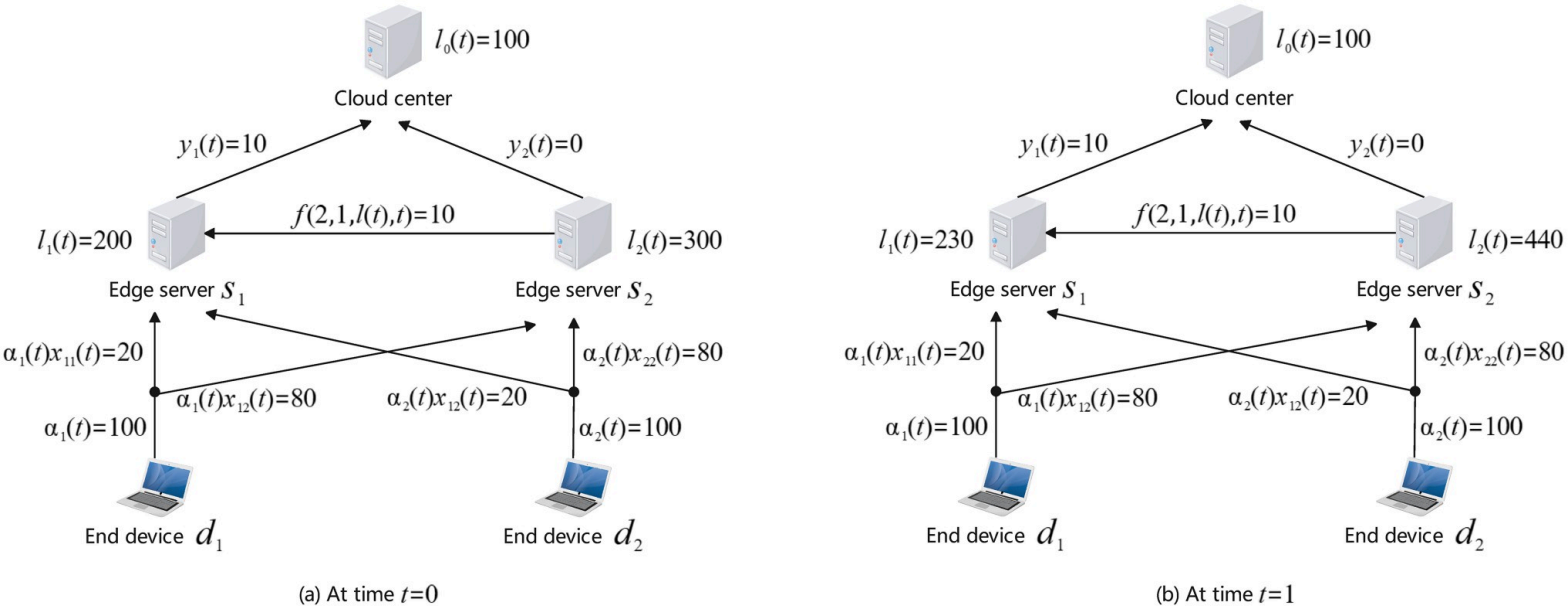
Simple numerical example for load evolution model.

To help understand the proposed load evolution model, we provide a simple numerical example as follows. Consider a tiny CEE system composed of only a cloud center, two edge servers and two device collections, as shown in Fig 5. Let a second be a unit time. Suppose the task executing rates are *β*_0_ = *β*_1_ = *β*_2_ = 10 tasks per second (tps). Suppose the tasks producing rates are *α*_1_(*t*) = *α*_2_(*t*) ≡ 100 tps. Suppose the initial load distribution is *L*_0_ = (*l*_0_(0), *l*_1_(0), *l*_2_(0)) = (100, 200, 300) tasks. Suppose the edge offloading strategy is *x*_11_(*t*) = *x*_21_(*t*) ≡ 0.2, *x*_12_(*t*) = *x*_22_(*t*) ≡ 0.8. Suppose the cloud offloading strategy is *y*_1_(*t*) ≡ 10, *y*_2_(*t*) ≡ 0 tps. Suppose the maximum of task migration rate is *f*_max_ = 10 tps, and the load balance scheme is presented by the function

$$\begin{array}{c} f\left(i,j,l\left(t\right),t\right)=\{\begin{array}{ccc} & 0, & \;\text{if}\;l_{i}\left(t\right)\geq l_{j}\left(t\right),\\ & f_{\text{max}}, & \;\text{if}\;l_{i}\left(t\right)<l_{j}\left(t\right). \end{array} \end{array}$$ (7)

Then, during the time interval [0, 1], there are *α*_1_(0)*x*_11_(0) = 20 tasks offloaded from the device *d*_1_ to the edge server *s*_1_, *α*_1_(0)*x*_12_(0) = 80 tasks from *d*_1_ to *s*_2_, *α*_2_(0)*x*_21_(0) = 20 tasks from *d*_2_ to *s*_1_, and *α*_2_(0)*x*_22_(0) = 80 tasks from *d*_2_ to *s*_2_. Meanwhile, because the edge server *s*_2_ has higher load than *s*_1_, i.e., *l*_2_(0) > *l*_1_(0), there are *f*(2, 1, *l*(0), 0) = 10 tasks migrated from *s*_2_ to *s*_1_ for load balance. Besides, there are *y*_1_(0) = 10 tasks offloaded from the edge server *s*_1_ to the cloud center. In addition, there are *β*_1_ = 10 tasks processed by the edge server *s*_1_, *β*_2_ = 10 tasks by *s*_2_.

As a consequence, during the time interval [0, 1], the load of the edge server *s*_1_ will change by

$$\begin{array}{c} l_{1}\left(1\right)-l_{1}\left(0\right)=\alpha_{1}\left(0\right)x_{11}\left(0\right)+\alpha_{2}\left(0\right)x_{21}\left(0\right)+f\left(2,1,l\left(0\right),0\right)-\beta_{1}-y_{1}\left(0\right)=230, \end{array}$$ (8)

the load of the edge server *s*_2_ will change by

$$\begin{array}{c} l_{2}\left(1\right)-l_{2}\left(0\right)=\alpha_{1}\left(0\right)x_{12}\left(0\right)+\alpha_{2}\left(0\right)x_{22}\left(0\right)-f\left(2,1,l\left(0\right),0\right)-\beta_{2}=440, \end{array}$$ (9)

and the load of the cloud center will change by

$$\begin{array}{c} l_{0}\left(1\right)-l_{0}\left(0\right)=y_{1}\left(0\right)-\beta_{0}=100. \end{array}$$ (10)

### 3.3 Latency model

Next, we need to establish a performance model to evaluate different CEE offloading strategies, so that we can have a criterion to select the optimal strategy from its feasible set.

In this paper, we consider the overall latency of the CEE system as a performance metric of CEE offloading strategies, as low latency is the most important feature of edge computing [30]. Specifically, we consider the following two types of latency: (i) task queuing latency occurred at edge servers and the cloud center; (ii) task propagation latency caused by edge offloading, cloud offloading, and the task migration for load balance.

**Fig 6:**
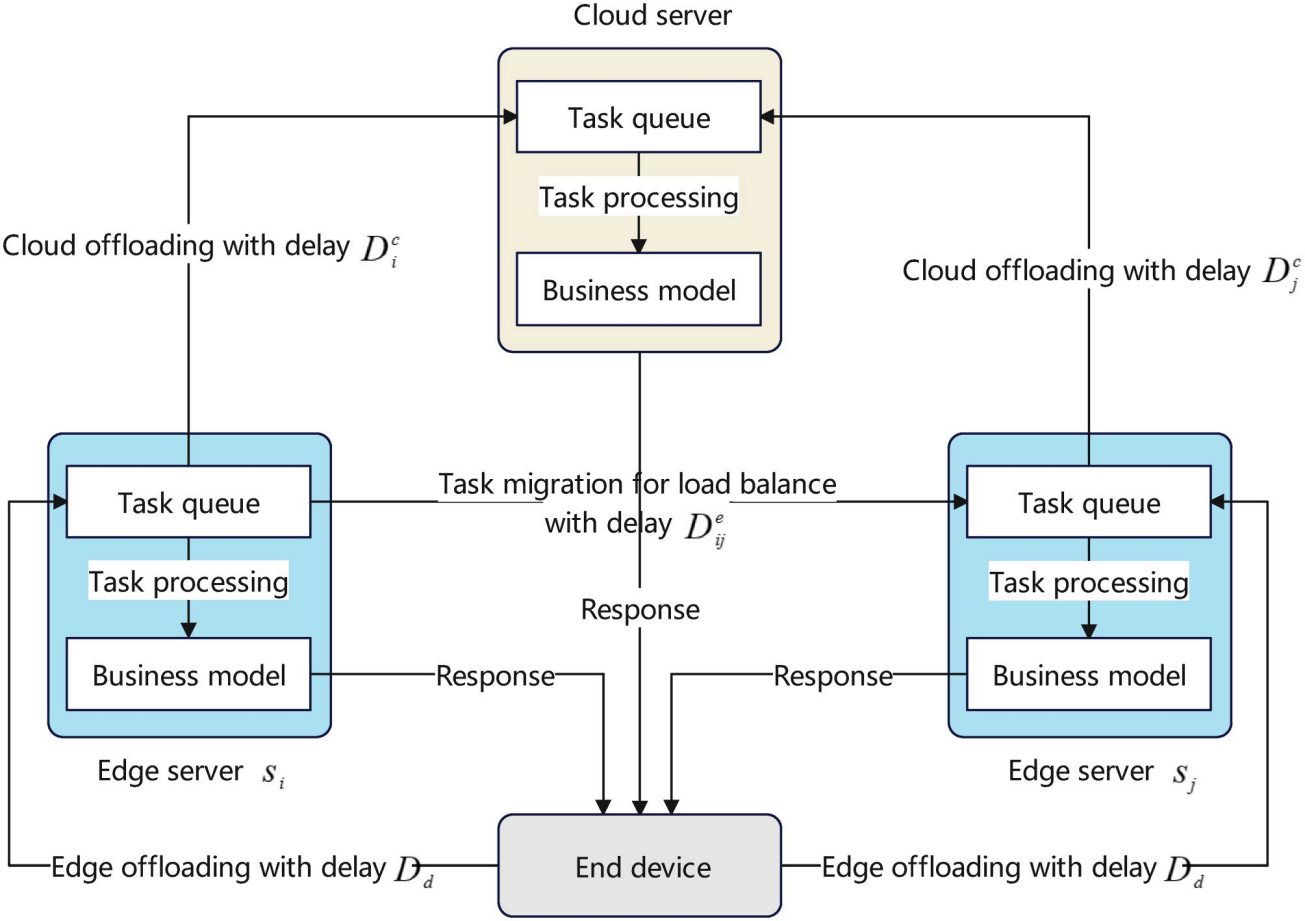
Simplified communication model of CEE systems.

To achieve that, let us consider the simplified communication model shown in Fig 6. Suppose each (cloud or edge) server contains a task queue and a business model responsible for handling tasks. Tasks arriving at a server will firstly be restored on the task queue and then be processed by the business model. Suppose the time interval [0, *T*] is short enough such that the network environment in this period is relatively stable. Denote *D*^*d*^ as the average propagation delay for offloading one task from an end device to an edge server through the access network, i.e., the time within which a task is transmitted from an end device to the task queue of an edge server. Denote $D_{ij}^{e}$ as the average propagation delay for migrating one task from the edge server *s*_*i*_ to the edge server *s*_*j*_ through the core network, i.e., the time within which a task is transmitted from the task queue of the server *s*_*i*_ to the task queue of the server *s*_*j*_. Denote $D_{j}^{c}$ as the average propagation delay for offloading one task from the edge server *s*_*j*_ to the cloud center through the Internet, i.e., the time within which a task is transmitted from the task queue of the server *s*_*j*_ to the task queue of the cloud server. Then, the overall latency of the CEE system is calculated in Theorem 2.

**Theorem 2** *Given a CEE offloading strategy* (**x**, *y*), *the overall latency of the CEE system is*

$$\begin{array}{c} \begin{array}{cc} J(x,y) & =\int_{0}^{T}\{D^{d}\sum_{i=1}^{N}\alpha_{i}\left(t\right)+\sum_{i=1}^{N}\sum_{j=1}^{M}D_{ij}^{e}f\left(i,j,l\left(t\right),t\right)+\sum_{j=0}^{M}l_{j}\left(t\right)\\ & \;\;\;+\sum_{j=1}^{M}D_{j}^{c}\Gamma \left(l_{j}\left(t\right)\right)y_{j}\left(t\right)\}dt. \end{array} \end{array}$$ (11)

**Proof 2** *Firstly, we quantify the task queuing latency. It is known that at time t, the queue length of the edge server s*_*j*_ is *l*_*j*_(*t*) *and that of the cloud center is l*_0_(*t*). *Thus, during the time horizon* [0, *T*], *the total time of all tasks staying in the queue of the server s*_*j*_ is $\int_{0}^{T}l_{j}\left(t\right)dt$ *and that of the cloud center is* $\int_{0}^{T}l_{0}\left(t\right)dt$. *Therefore, the overall queuing latency of the CEE system in* [0, *T*] *is*

$$\begin{array}{c} J_{Q}=\int_{0}^{T}\sum_{j=0}^{M}l_{j}\left(t\right)dt. \end{array}$$ (12)

*Secondly, we quantify the task propagation latency. At time t*, *tasks are offloaded from end devices to edge servers at the total rate of* $\sum_{i=1}^{N}\alpha_{i}\left(t\right)$. *Therefore, during the time interval* [0, *T*], *the total latency of edge offloading is*

$$\begin{array}{c} J_{T}^{E}=D^{d}\int_{0}^{T}\sum_{i=1}^{N}\alpha_{i}\left(t\right)dt. \end{array}$$ (13)

*Also, at time t*, *tasks are offloaded from the edge server s*_*j*_ *to the cloud center at the rate of* Γ(*l*_*j*_(*t*))*y*_*j*_(*t*). *Hence, in the time interval* [0, *T*], *the total latency of cloud offloading is*

$$\begin{array}{c} J_{T}^{C}=\sum_{j=1}^{M}D_{j}^{c}\int_{0}^{T}\Gamma \left(l_{j}\left(t\right)\right)y_{j}\left(t\right)dt. \end{array}$$ (14)

*In addition, at time t*, *tasks in the edge server s*_*i*_ *are migrated to the server s*_*j*_ *at the rate of f*(*i*, *j*, *l*(*t*), *t*). *Accordingly, in the time interval* [0, *T*], *the total latency of task migration is*

$$\begin{array}{c} J_{T}^{B}=\sum_{i=1}^{N}\sum_{j=1}^{M}D_{ij}^{e}\int_{0}^{T}f\left(i,j,l\left(t\right),t\right)dt. \end{array}$$ (15)

*Combining the above discussions, we obtain the result by directly calculating*

$$\begin{array}{c} J\left(x,y\right)=J_{Q}+J_{T}^{E}+J_{T}^{C}+J_{T}^{B}. \end{array}$$ (16)

*The proof is complete*.

We refer to Eq (11) as the *latency model*. A better CEE offloading strategy should result in less latency.

### 3.4 Optimal control problem

Now, we are ready to formulate an optimization model to describe the LBAO problem, with the CEE offloading strategy as the decision variable, the latency model (11) as the objective functional, and the load evolution model (2) as a constraint.

$$\begin{array}{c} \begin{array}{cc} & \underset{(x,y)\in \Omega }{\text{min}}\;J\left(x,y\right)\\ s.t.\; & l(t)\;\text{satisfies}\;\text{the}\;\text{load}\;\text{evolution}\;\text{model}\;\text{(}2\text{)}. \end{array} \end{array}$$ (17)

The above optimization problem is a continuous-time optimal control problem [31]. After solving it, we will attain the CEE offloading strategy that minimizes the overall latency of the CEE system.

## 4 Solution

In this section, we discuss the solution to the optimal control model (17). First, we analyze the feasibility of some typical numerical methods in solving the model (17). Second, we apply GA to solve the model (17). Third, we make a rough analysis of the time complexity of GA in solving the model (17).

### 4.1 Analysis of feasible numerical methods

Recall that the problem (17) is a continuous-time optimal control model. As the survey [32] reports, numerical methods to solve a continuous-time optimal control problem can be divided into two categories: indirect methods and direct methods. In an indirect method, a set of necessary conditions of optimality will be derived and then the oringinal optimal control problem will be equivalently transformed to a multi-point boundary value (MPBV) problem [33]. On this basis, a proper iterative algorithm will be developed to solve the MPBV problem, and a candidate optimal solution satisfying all the known necessary conditions of optimality will be attained. When the original optimal control problem is extremely simple, indirect methods can perform well with regard to solution accuracy and algorithm complexity. However, if the original optimal control problem is high-dimensional and complex, it would be very difficult to guess a proper initial solution for iteratively solving the MPBV problem. Thus, indirect methods are less common than direct methods [34].

Contrary to indirect methods, direct methods suggest treating optimal control problems as nonlinear optimization problems. In a direct method, time-varying decision variables will be represented (or approximated) by some kind of static parameters, and then the original optimal control problem will be addressed by using well-known optimization techniques. Compared with indirect methods, direct methods do not rely on the optimality necessary conditions of the original optimal control problem and is therefore more versatile in practical applications. Thus, in this paper, we intend to address the optimal control model (17) through a direct method.

The survey [32] also points out that direct methods can be further classified into two categories: nonlinear programming (NLP) -based methods and heuristic-based methods. For simple optimal control models or the cases where the control and state variables are low dimensional, NLP-based methods can achieve good performance. However, NLP-based methods may not be suitable to solve the optimal control model (17) due to the heavy dependence on function approximation. Specifically, with regard to the optimal control model (17), NLP-based methods require using four interpolation polynomials to approximate the strategies *x*(*t*) and *y*(*t*), the differential equations of the load evolution trajectory *l*(*t*), and the definite integration of the latency model *J*. Because polynomial interpolation will inevitably produce errors, the solution of the approximate problem would deviate from the solution of the original optimal control problem (17). Thus, an appropriate method should reduce the dependence on function approximation as much as possible.

Fortunately, heuristic-based methods, which require only the approximation for the decision functions *x*(*t*) and *y*(*t*), provide a promising way to solve the LBAO problem. Therefore, in this paper we solve the optimal control model (17) through a heuristic-based method. GA [9] is a commonly used meta-heuristic method to find satisfactory solutions to a complex optimization problem. As the performance of GA has been widely proven, in the subsequent subsections, we apply GA to solve the optimal control problem (17). Specifically, we proceed into the following four steps. First, design an encoding scheme to represent CEE offloading strategies by *chromosomes* (i.e., a set of parameters), and design a decoding scheme to *safely* map chromosomes to their corresponding CEE offloading strategies, so as to ensure the resulting CEE offloading strategies must satisfy the feasible set (1). Second, design an initialization scheme to generate random feasible chromosomes. Third, design a fitness evaluation scheme to select high-quality chromosomes. Finally, design a crossover-mutation operator to produce better chromosomes from the existing ones. The details are as follows.

### 4.2 Encoding and decoding

An encoding scheme is responsible for transferring a CEE offloading strategy to a set of controllable parameters called a chromosome. In this paper, we use the widely adopted Legendre-Gauss (LG) method [35], which has been proven to be an accurate and efficient discretization technique. Specifically, we proceed into the following three steps.

First, we map the time interval *t* ∈ [0, *T*] to *τ* ∈ [−1, 1] by

$$\begin{array}{c} \tau =\frac{2}{T}t-1,\;t=\frac{T}{2}\tau +\frac{T}{2}. \end{array}$$ (18)

Second, we determine an integer *n* and select *n* collocation points [35] by solving the roots of the equation *P*_*n*_(*τ*) = 0, where *P*_*n*_(*τ*) is the Legendre polynomial [36]. Denote the resulting *n* collocation points by (*τ*_1_, …, *τ*_*n*_).

Third, we approximate the CEE offloading strategy (**x**, *y*) by

$$\begin{array}{c} \left\{ \begin{array}{cc} x_{ij}\left(\tau \right) & \approx X_{ij}\left(\tau \right)=\sum_{k=1}^{n}x_{ij}\left(\tau_{k}\right)L_{k}\left(\tau \right),\;i=1,\cdots ,N,\;j=1,\cdots ,M,\\ y_{j}\left(\tau \right) & \approx Y_{j}\left(\tau \right)=\sum_{k=1}^{n}y_{j}\left(\tau_{k}\right)L_{k}\left(\tau \right),\;j=1,\cdots ,M, \end{array}\right. \end{array}$$ (19)

where *L*_*k*_(*τ*) are the Lagrange polynomials [37]

$$\begin{array}{c} L_{k}\left(\tau \right)=\prod_{m=0,m\neq k}^{n}\frac{\tau -\tau_{m}}{\tau_{k}-\tau_{m}},\;k=1,\cdots ,n. \end{array}$$ (20)

It follows from observation that *x*_*ij*_(*τ*) = *X*_*ij*_(*τ*) and *y*_*j*_(*τ*) = *Y*_*j*_(*τ*) hold true for all the collocation points *τ* = *τ*_1_, …, *τ*_*n*_. This way, the approximate CEE offloading strategy (**X**, *Y*) is determined by all the fixed points *x*_*ij*_(*τ*_*k*_) and *y*_*j*_(*τ*_*k*_), where *i* = 1, …, *N*, *j* = 1, …, *M*, *k* = 1, …, *n*.

Therefore, we define a chromosome as an [(*N* + 1) × (*M* × *n*)]-dim matrix

$$\begin{array}{c} c=[\begin{array}{cccccccc} x_{11}\left(\tau_{1}\right) & \cdots & x_{11}\left(\tau_{n}\right) & x_{12}\left(\tau_{1}\right) & \cdots & x_{12}\left(\tau_{n}\right) & \cdots & x_{1M}\left(\tau_{n}\right)\\ \vdots & & & & & & & \vdots \\ x_{N1}\left(\tau_{1}\right) & \cdots & x_{N1}\left(\tau_{n}\right) & x_{N2}\left(\tau_{1}\right) & \cdots & x_{N2}\left(\tau_{n}\right) & \cdots & x_{NM}\left(\tau_{n}\right)\\ y_{1}\left(\tau_{1}\right) & \cdots & y_{1}\left(\tau_{n}\right) & y_{2}\left(\tau_{1}\right) & \cdots & y_{2}\left(\tau_{n}\right) & \cdots & y_{M}\left(\tau_{n}\right) \end{array}]. \end{array}$$ (21)

After designing the encoding scheme, we need a decoding scheme to map a chromosome to its corresponding CEE offloading strategy. As the encoding scheme is an approximate mapping, a challenge in decoding is to ensure the resulting CEE offloading strategy must satisfy the feasible set (1). To this end, a heuristic decoding scheme is shown in Algorithm 1. The main idea of this heuristic algorithm is straightforward. First, we forcefully make the parts of **x** and *y* that exceed their upper and lower bounds become their upper and lower bounds. Second, for the parts of **x** that do not satisfy the conditions $\sum_{m=1}^{M}x_{im}\left(t\right)=1$, we modify these parts through normalization.

**Algorithm 1 Decoding**

**Input:** A chromosome **c**.

**Output:** A CEE offloading strategy (**x**, *y*).

1: // Below, calculate the approximate functions.

2: Calculate (**X**, *Y*) with the chromosome **c** from Eqs (19) and (21).

3: // Below, ensure the conditions 0 ≤ *x*_*ij*_(*t*) ≤ 1, ∀*i*, *j*.

4: **for** *i* ← 1: *N*

5:  **for** *j* ← 1: *M*

6:   ${\tilde{x}}_{ij}\left(t\right)\leftarrow \text{min}\left\{1,\text{max}\left\{0,X_{ij}\left(t\right)\right\}\right\},\;0\leq t\leq T$.

7: // Below, ensure the conditions $\sum_{m=1}^{M}x_{im}\left(t\right)=1$, **for** *alli*.

8: **for** *i* ← 1: *N*

9:  **if** $\sum_{k=1}^{M}{\tilde{x}}_{ik}\left(t\right)=1$

10:   **for** *j* ← 1: *M*

11:    $x_{ij}\left(t\right)\leftarrow {\tilde{x}}_{ij}\left(t\right),\;0\leq t\leq T$.

12:  **else**

13:   **for** *j* ← 1: *M* **do**

14:    $x_{ij}\left(t\right)\leftarrow \frac{{\tilde{x}}_{ij}\left(t\right)}{\sum_{k=1}^{M}{\tilde{x}}_{ik}\left(t\right)},\;0\leq t\leq T$.

15: // Below, ensure the conditions 0 ≤ *y*_*j*_(*t*) ≤ *y*_max_, **for** *allj*.

16: **for** *j* ← 1: *M* **do**

17:  *y*_*j*_(*t*) ← min{*y*_max_, max{0, *Y*_*j*_(*t*)}}, 0 ≤ *t* ≤ *T*.

18: // This way, (**x**, *y*) must satisfy the feasible set (1). Thus, return it as a result.

19: **return** (**x**, *y*).

### 4.3 Initialization

An initialization scheme is responsible for generating random chromosomes. As [38] points out, what is the most important in this step is to maintain good diversity of the generated chromosomes to prevent *premature convergence*. Thus, it is needed to make the generated chromosomes uniformly distributed throughout their feasible set. To this end, an initialization scheme is displayed in Algorithm 2.

**Algorithm 2 Initialization**

**Input:** Not applicable.

**Output:** A random chromosome **c**.

1: // Below, consider the expression of **c** in Eq (21).

2: **for** *k* ← 1: *n* **do**

3:  **for** *i* ← 1: *N* **do**

4:   Generate a *M*-dim random vector from [0, 1]^*M*^. Denote it by **r**.

5:   **for** *j* ← 1: *M*

6:    $x_{ij}\left(\tau_{k}\right)\leftarrow \frac{r_{j}}{\sum_{m=1}^{M}r_{m}}$.

7:  Generate a *M*-dim random vector from [0, *y*_max_]^*M*^. Denote it by **r**.

8:  **for** *j* ← *M*

9:   *y*_*j*_(*τ*_*k*_) ← *r*_*j*_.

10: **return** **c**.

### 4.4 Fitness evaluation

A fitness evaluation scheme aims to measure the quality of different chromosomes. The higher the fitness, the better the chromosome. In this paper, we simply define the fitness of a chromosome by the negative number of the latency it causes. The detail of the fitness evaluation scheme is given in Algorithm 3, whose main idea is straightforward.

**Algorithm 3 Fitness**

**Input:** A chromosome **c**.

**Output:** The fitness value of the input chromosome.

1: (**x**, *y*) ← Decoding(**c**).

2: Calculate *l* with (**x**, *y*) based on the load evolution model (2).

3: Calculate the latency *J*(**x**, *y*) by the latency model (11).

4: **return** −*J*(**x**, *y*).

### 4.5 Crossover and mutation

A crossover-mutation operator is responsible for producing chromosomes with higher quality from the existing ones. In this paper, we adopt the standard uniform crossover method [39] and the standard uniform mutation method [39]. A pseudo-code for this step is given in Algorithm 4.

**Algorithm 4 Evolution**

**Input:** A pair of chromosomes (**c**^(1)^, **c**^(2)^), crossover probability *p*_*c*_, and mutation probability *p*_*m*_.

**Output:** Two new chromosomes.

1: **for** *i* ← 1: (*N* + 1) **do**

2:  **for** *j* ← 1: (*M* × *n*) **do**

3:   // Crossover

4:   Generate a random number *r* from [0, 1].

5:   **if** *r* < *p*_*c*_

6:    Swap $c_{ij}^{\left(1\right)}$ and $c_{ij}^{\left(2\right)}$.

7:   // Mutation.

8:   **for** *q* ← 1: 2

9:    Generate a random number *r* from [0, 1].

10:    **if** *r* < *p*_*m*_

11:     **if** *i* = *N* + 1

12:      Set $c_{ij}^{\left(q\right)}$ by a random value from [0, *y*_max_].

13:     **else**

14:      Set $c_{ij}^{\left(q\right)}$ by a random value from [0, 1].

15: // Check if $\sum_{j=1}^{M}x_{ij}\left(\tau_{k}\right)=1$. If not, modify the related elements.

16: **for** *q* ← 1: 2 **do**

17:  **for** *i* ← 1: *N* **do**

18:   **for** *k* ← 1: *n* **do**

19:    **if** $\sum_{j=1}^{M}x_{ij}^{\left(q\right)}\left(\tau_{k}\right)\neq 1$

20:     **for** *j* ← 1: *M*

21:      $x_{ij}^{\left(q\right)}\left(\tau_{k}\right)\leftarrow \frac{x_{ij}^{\left(q\right)}\left(\tau_{k}\right)}{\sum_{m=1}^{M}x_{im}^{\left(q\right)}\left(\tau_{k}\right)}$.

22: **return** (**c**^(1)^, **c**^(2)^)

### 4.6 Overview of LBAO algorithm

**Fig 7:**
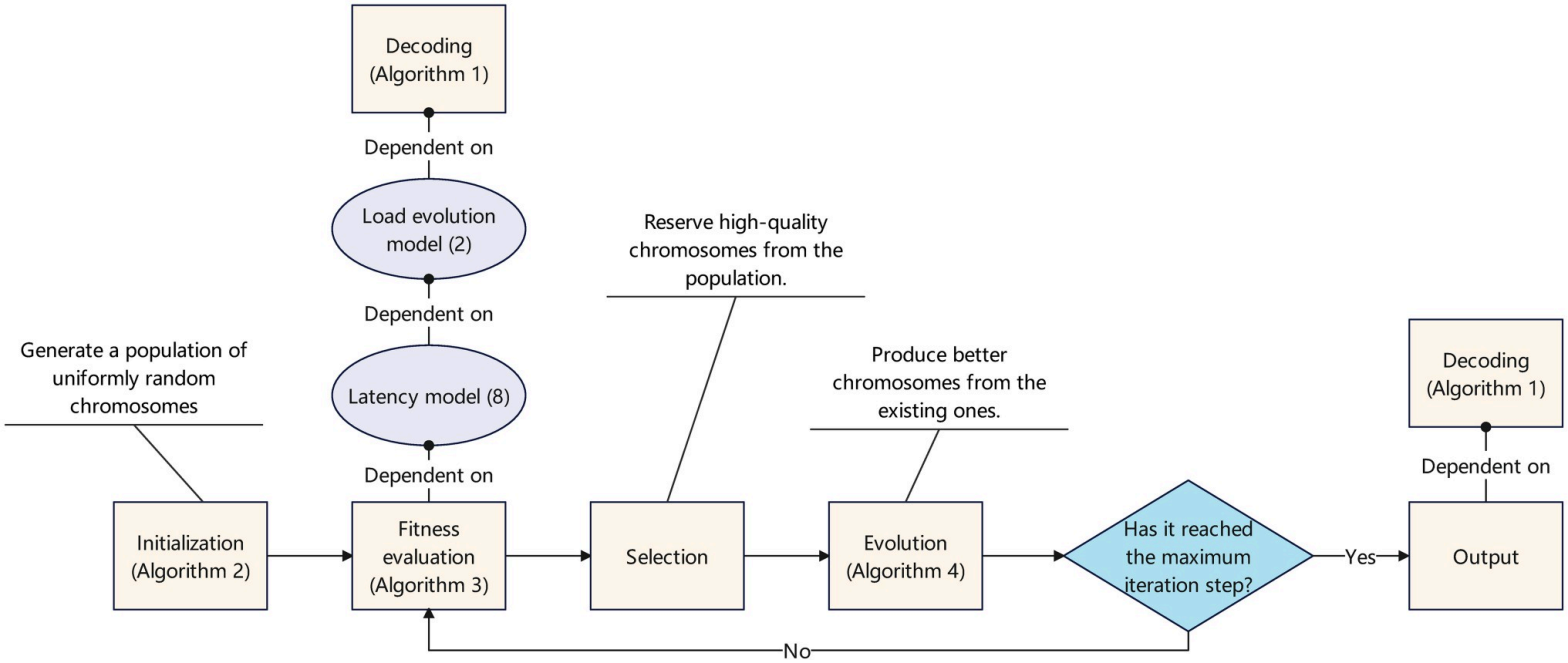
Flow chart of LBAO algorithm.

According to the above discussions, a overall procedure of our GA-based method is presented in Algorithm 5. We refer to it as the *LBAO algorithm*. Also, a flow chart illustrating the LBAO algorithm is shown in Fig 7. In addition, the source codes of the LBAO algorithm have been uploaded to GITHUB, whose link is [40]. Through the LBAO algorithm, we can obtain a satisfactory CEE offloading strategy.

**Algorithm 5 LBAO**

**Input:** Population size *N*_*P*_, crossover probability *p*_*c*_, mutation probability *p*_*m*_, and maximal iteration step *Q*.

**Output:** A satisfactory CEE offloading strategy (x*, y*).

1: // Population initialization and fitness evaluation

2: **for** *i* ← 1: *N*_*P*_ **do**

3:  **c**^(*i*)^ ← Initialization().

4:  *F*(**c**^(*i*)^) ← Fitness(**c**^(*i*)^).

5: // Population evolution

6: **for** *q* ← 1: *Q* **do**

7:  // Select chromosomes using the standard roulette-wheel operator [39].

8:  $\left(c^{\left(1\right)},\ldots ,c^{\left(N_{P}\right)}\right)$ ← RouletteWheel(*F*_1_, …, *F*_*NP*_).

9:  // Crossover and mutation

10:  **for** *i* ← 1: 2: *N*_*P*_ **do**

11:   (**c**^(*i*)^, **c**^(*i*+1)^) ← Evolution(**c**^(*i*)^, **c**^(*i*+1)^, *p*_*c*_, *p*_*m*_).

12: // Fitness evaluation for the new generation of population.

13:  **for** *i* ← 1: *N*_*P*_ **do**

14:   *F*(**c**^(*i*)^) ← Fitness(**c**^(*i*)^).

15: // Find the best chromosome from the population, and return its strategy

16: $c^{*}\leftarrow \arg_{c=c^{\left(1\right)},\ldots ,c^{\left(N_{P}\right)}}F(c)$.

17: (x*, y*) ← Decoding(c*).

18: **return**(x*, y*)

### 4.7 Time complexity of LBAO algorithm

Next, let us make a rough analysis of the time complexity of the proposed LBAO algorithm. Denote the average time consumption of the initialization, fitness evaluation, and evolution operators by *C*_*I*_, *C*_*F*_, and *C*_*E*_, respectively. Then, by directly observing the LBAO algorithm shown in Algorithm 5, we can obtain that the total time consumption is approximately equal to

$$\begin{array}{c} \begin{array}{cc} C_{total} & =N_{P}\left(C_{I}+C_{F}\right)+Q(\frac{N_{P}}{2}C_{E}+N_{P}C_{F})\\ & =N_{P}C_{I}+N_{P}\left(1+Q\right)C_{F}+\frac{QN_{P}}{2}C_{E}. \end{array} \end{array}$$ (22)

In practice, the initialization, fitness evaluation, and evolution operators are all implemented by matrix-based operations (e.g., matrix multiplication). Thus, we define matrix-based operations as basic operations and denote by *C* the average time consumption of a basic operation. Then, by direct observation, we can obtain that *C*_*I*_ ≈ *C*, *C*_*E*_ ≈ 2*C*. Next, let us estimate the time consumption of the fitness evaluation operator. As shown in Algorithm 3, it mainly contains the calculation of the load evolution model (2) and latency model (11), which are essentially differential equations and a function integration, respectively. In this paper, we use the Euler method [41] (an efficient numerical algorithm to solve differential equations and function integration) to calculate the load evolution model and latency model. When the Euler method is applied, the total time horizon [0, *T*] is uniformly divided into *N*_*T*_ small time intervals, and for each time interval, the derivative of every function is calculated through only one matrix-based operation. Because the load evolution model contains (*M* + 1) functions *l*_*j*_ and the latency model contains just one function *J*, the time consumption of the fitness evaluation operator is *C*_*F*_ = [(*M* + 1) + 1]*N*_*T*_*C*.

Combining the above discussions, it follows that

$$\begin{array}{c} \begin{array}{cc} C_{total} & =N_{P}C+N_{P}\left(1+Q\right)\left(M+2\right)N_{T}C+\frac{1}{2}QN_{P}2C\\ & =N_{P}\left(Q+1\right)\left[\left(M+2\right)N_{T}+1\right]C. \end{array} \end{array}$$ (23)

From Eq (23), we can see that the time consumption of the LBAO algorithm is determined by the population size *N*_*P*_, the maximum iteration step *Q*, and the discrete precision *N*_*T*_, provided that the number of edge servers *M* is given by reality conditions. In practice, the population size *N*_*P*_ and maximum iteration step *Q* will affect the optimality of the GA-based LBAO algorithm, whereas the discrete precision *N*_*T*_ will affect the precision of the Euler-based numerical result. Thus, if we intend to obtain an offloading strategy with better quality, we may set the parameters *N*_*P*_, *Q*, *N*_*T*_ by large values but the total time consumption of running the LBAO algorithm will increase. Otherwise, if we intend to apply the LBAO algorithm to real-time scenarios, we may set the parameters *N*_*P*_, *Q*, *N*_*T*_ by small values such that the time consumption is low enough (though doing so may lead to some sacrifices in the quality of the results).

## 5 Numerical experiment

In the previous section, we proposed the LBAO algorithm based on the GA framework. In this section, we are devoted to verifying the LBAO algorithm. First, we describe the parameters used in our numerical experiments. Second, we investigate the optimal configuration of the LBAO algorithm. Third, we examine the performance of the LBAO algorithm. Finally, we give insight into the influence of load balance in CEE offloading.

### 5.1 Parameter setting

In our numerical experiments, we consider a CEE system composed of a remote cloud server, two edge servers, and three end-device collections. Thus, let *M* = 2, *N* = 3. Let *T* = 6 seconds. As the propagation delay between end devices and edge servers can range from 30 milliseconds (ms) to 40 ms [42], we set *D*^*d*^ = 35 ms by the average value. In addition, as the propagation delay between edge servers can range from 0.1 ms [43] to 5 ms [44], we set each $D_{ij}^{e}$ randomly within the range [0.1, 5]. Besides, we assume that the propagation delay between edge servers and cloud centers can range from 100 ms to 500 ms (without network congestion), and we set each $D_{j}^{c}$ randomly within the range [100, 500].

Next, we suppose the load balance scheme is supported by the Round Robin (RR) algorithm [45], one of the most widely used load balance algorithms. In the RR scheme, tasks from an edge server are equally dispatched to all the idle servers. Suppose the maximum of task migration rate is *f*_max_ = 200 tasks per second (tps). Following [25], the load balance function *f* can be defined as

$$\begin{array}{c} f\left(i,j,l\left(t\right),t\right)=\left\{ \begin{array}{ccc} & 0,\; & \text{if}\;l_{i}\left(t\right)\leq l_{j}\left(t\right),\\ & f_{\text{max}},\; & \text{if}\;l_{i}\left(t\right)>l_{j}\left(t\right). \end{array}\right. \end{array}$$ (24)

Moreover, we suppose the task processing rate of each edge server can range from 2000 to 3000 tps, and that of the cloud center is 3000 tps. Thus, we set *β*_0_ = 3000 and set each *β*_*j*_, *j* = 1, …, *M*, randomly within the range [2000, 3000]. To present a case of flash crowd (i.e., the network congestion which occurs when a huge number of end devices request an edge server simultaneously), we set the edge offloading rates by *α*_*i*_(*t*) = 6000*i* tps. Besides, let the maximum cloud offloading rate be *y*_max_ = 200 tps.

### 5.2 Optimal configuration of LBAO algorithm

Having described the parameters used in our experiments, let us investigate the optimal configuration of the LBAO algorithm. Because the LBAO algorithm is based on the GA framework, there are three crucial parameters that can affect the quality of the resulting solution: the population size *N*_*P*_, the crossover probability *p*_*c*_, and the mutation probability *p*_*m*_. Below, we determine these three parameters by setting them by different values and examining their resulting algorithm performance.

Let *N*_*P*_ ∈ {120, 240, 360}, *p*_*c*_ ∈ {0.05, 0.10, …, 0.50}, *p*_*m*_ ∈ {0.025, 0.050, …, 0.100}. Then, we run the LBAO algorithm for each parameter combination with the same iteration steps and compare the results. Denote J* as the optimal latency yielded from the LBAO algorithm. Then, Tables 1–3 display the algorithm performance under different parameter combinations with regard to *N*_*P*_ = 120, *N*_*P*_ = 240, and *N*_*P*_ = 360, respectively. From these three tables, it is seen that when *N*_*P*_ = 360, *p*_*c*_ = 0.50, *p*_*m*_ = 0.075, the LBAO algorithm can achieve the best performance as the optimal latency is J* = 182.839 thousands seconds. Thus, we recommend *N*_*P*_ = 360, *p*_*c*_ = 0.50, *p*_*m*_ = 0.075 as the optimal configuration of the LBAO algorithm with respect to the case we examine.

**Table 1:** Optimal latency under different crossover and mutation probabilities when *N*_*P*_ = 120.

*p*_*c*_	0.05	0.10	0.15	0.20	0.25	0.30	0.35	0.40	0.45	0.50
J*										
*p*_*m*_										
0.025	183.416	183.429	183.091	183.210	182.957	183.282	182.904	183.072	182.874	183.150
0.050	183.216	183.039	182.978	183.028	182.880	183.032	182.978	182.960	183.020	182.877
0.075	183.396	182.963	183.109	182.952	183.059	182.876	182.989	183.039	182.885	183.158
0.100	183.346	182.889	182.984	183.078	182.904	**182.869**	182.962	183.255	182.901	183.110

**Table 2:** Optimal latency under different crossover and mutation probabilities when *N*_*P*_ = 240.

*p*_*c*_	0.05	0.10	0.15	0.20	0.25	0.30	0.35	0.40	0.45	0.50
J*										
*p*_*m*_										
0.025	183.021	183.109	182.872	182.902	182.890	182.955	182.881	182.900	182.885	182.903
0.050	182.946	182.924	183.074	182.851	182.887	183.177	182.876	182.973	183.068	182.870
0.075	183.003	183.064	182.972	182.986	182.851	**182.850**	182.898	183.033	182.870	183.188
0.100	183.252	183.015	183.338	182.875	182.857	182.876	182.888	182.855	182.950	182.942

**Table 3:** Optimal latency under different crossover and mutation probabilities when *N*_*P*_ = 360.

*p*_*c*_	0.05	0.10	0.15	0.20	0.25	0.30	0.35	0.40	0.45	0.50
J*										
*p*_*m*_										
0.025	182.961	183.038	182.940	183.009	183.334	182.851	182.862	182.848	182.909	182.886
0.050	183.007	182.993	182.903	182.881	182.841	182.945	182.973	182.852	182.851	182.928
0.075	183.228	182.892	182.873	182.850	182.880	182.845	182.907	182.847	182.835	**182.839**
0.100	183.316	183.062	182.846	182.949	182.859	182.852	182.855	182.921	182.897	182.872

### 5.3 Effectiveness of LBAO algorithm

Though the GA framework is considered effective in tackling most optimization problems [39], there is still a need to verify if the solution obtained from the GA-based LBAO algorithm achieves a satisfactory performance. To this end, we intend to compare the LBAO solution with a large number of randomly generated CEE offloading strategies and verify if the performance of the LBAO solution is better than the best performance among all the random CEE offloading strategies. The reason for this experiment approach is straightforward. According to the Monte Carlo theory [46], with the increase of the number of randomly generated CEE offloading strategies, the best performance among all the random strategies will gradually approach the global optimal value. Thus, when the number of random strategies is large enough and the LBAO solution is better than all the random strategies, it is reasonable to recommend the LBAO solution as a satisfactory result.

Let *N*_*R*_ denote the number of randomly generated CEE offloading strategies. Then, a comparison between the LBAO algorithm and the Monte Carlo method is shown in Table 4. From this table, it is seen that the optimal latency of random CEE offloading strategies decreases with the increase of the number of random CEE offloading strategies, which satisfies the statement of the Monte Carlo theory. However, even though the number of random strategies is as large as *N*_*R*_ = 10, 000, 000, the optimal latency of random strategies is much higher than that of the LBAO solution. Besides, all the numerical experiments are conducted on the same PC machine with an AMD 5800X CPU and 32 GB memory, and it is seen that the LBAO algorithm is much more efficient than the Monte Carlo method as the runtime of the LBAO algorithm is far less than that of the Monte Carlo method. Thus, the LBAO algorithm is effective.

**Table 4:** Comparison between LBAO algorithm and Monte Carlo (MC) method.

Method	MC	MC	MC	MC	MC	MC	MC	LBAO
*N*_*R*_	1,000	10,000	100,000	1,000,000	2,000,000	5,000,000	10,000,000	N/A
J*	198.031	197.790	197.304	197.035	197.035	197.013	197.011	**182.839**
Runtime	3.18s	33.29s	5m 18s	56m 27s	1h 52m	3h 48m	9h 42m	**12.45s**

In addition, we compare the LBAO algorithm with other methods, including but not limited to those applied to industrial IoT scenarios. Let us introduce several baseline methods as follows.

- Cloud Horizon (ClHo) scheme [6]. At any time, each end device selects a random edge server to offload all computational tasks and each edge server always performs the cloud offloading with the maximum capability. With respect to our load evolution model, the offloading strategy is configured by $x_{ij}\left(t\right)\equiv \frac{1}{M}$, ∀*i*, *j*, *y*_*j*_(*t*) ≡ *y*_max_, ∀*j*.
- First Come First Service (FCFS) scheme [19]. At any time, each task produced by end devices is offloaded to edge servers in sequence, following the first come first service principle. Similarly, each task on the queue of an edge server is offloaded to the cloud center in sequence, following the first come first service principle. With regard to our load evolution model, the offloading strategy is configured by $x_{ij}\left(t\right)\equiv \frac{1}{M}$, ∀*i*, *j*, $y_{j}\left(t\right)=\text{min}\left\{y_{\text{max}},\frac{l_{j}\left(t\right)}{2}\right\}$, ∀*j*.
- Edge Processing Only (EPS) scheme [47]. At any time, each end device can only select a random edge server to offload all computational tasks and the cloud offloading is invalid. With respect to our load evolution model, the offloading strategy is configured by $x_{ij}\left(t\right)\equiv \frac{1}{M}$, ∀*i*, *j*, *y*_*j*_(*t*) ≡ 0, ∀*j*.

**Fig 8:**
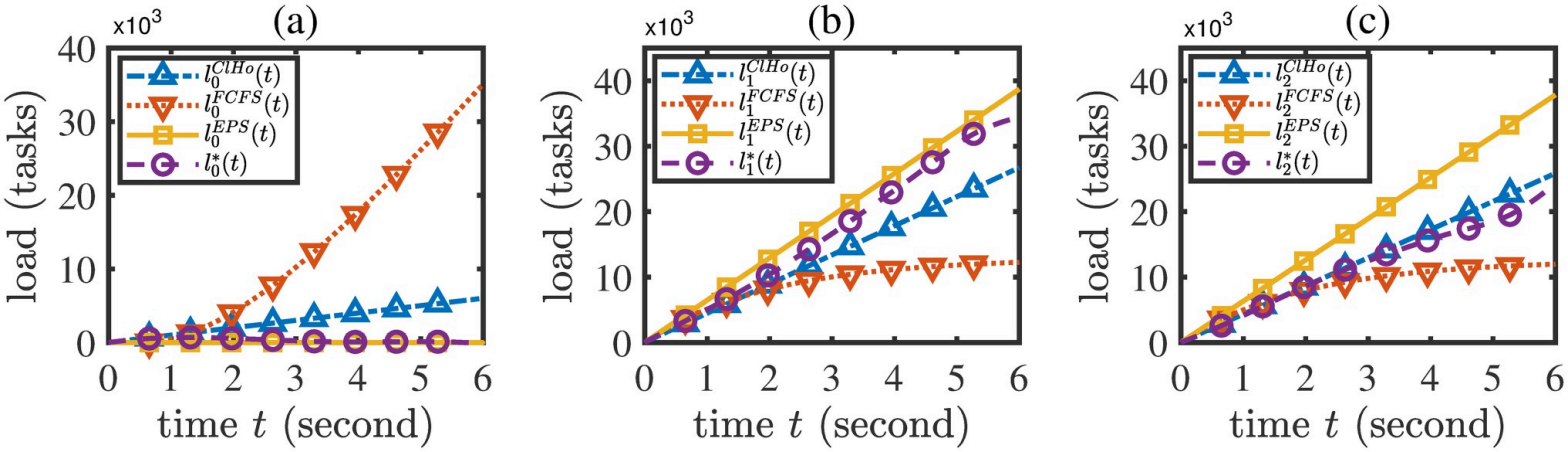
Comparison between the load evolution trajectories *l*^*ClHo*^, *l*^*FCFS*^, *l*^*EPS*^ and l*.

A performance comparison of the ClHo, FCFS, EPS, MC, LBAO methods is shown in Table 5, from which it is seen that the proposed LBAO algorithm is better than the four baseline methods in terms of task total latency. In addition, denote the load evolution trajectories under the ClHo, FCFS, EPS schemes by *l*^*ClHo*^, *l*^*FCFS*^, *l*^*EPS*^, respectively. Then, the load evolution trajectories under the LBAO algorithm and the ClHo, FCFS, EPS methods are compared in Fig 8. From this figure, it is seen that the cloud load under the LBAO algorithm is much lower than those under the baseline methods. This result implies that in the current network environment, the propagation delay for cloud offloading may be relatively high. Thus, the LBAO strategy does not recommend the excessive use of cloud offloading, but rather suggests fully utilizing the potential capability brought by the load balance between edge servers.

**Table 5:** Comparison of different methods in terms of total latency.

Strategy	ClHo	FCFS	EPS	MC	LBAO
Total latency	192.834	191.863	233.287	197.011	**182.839**

**Fig 9:**
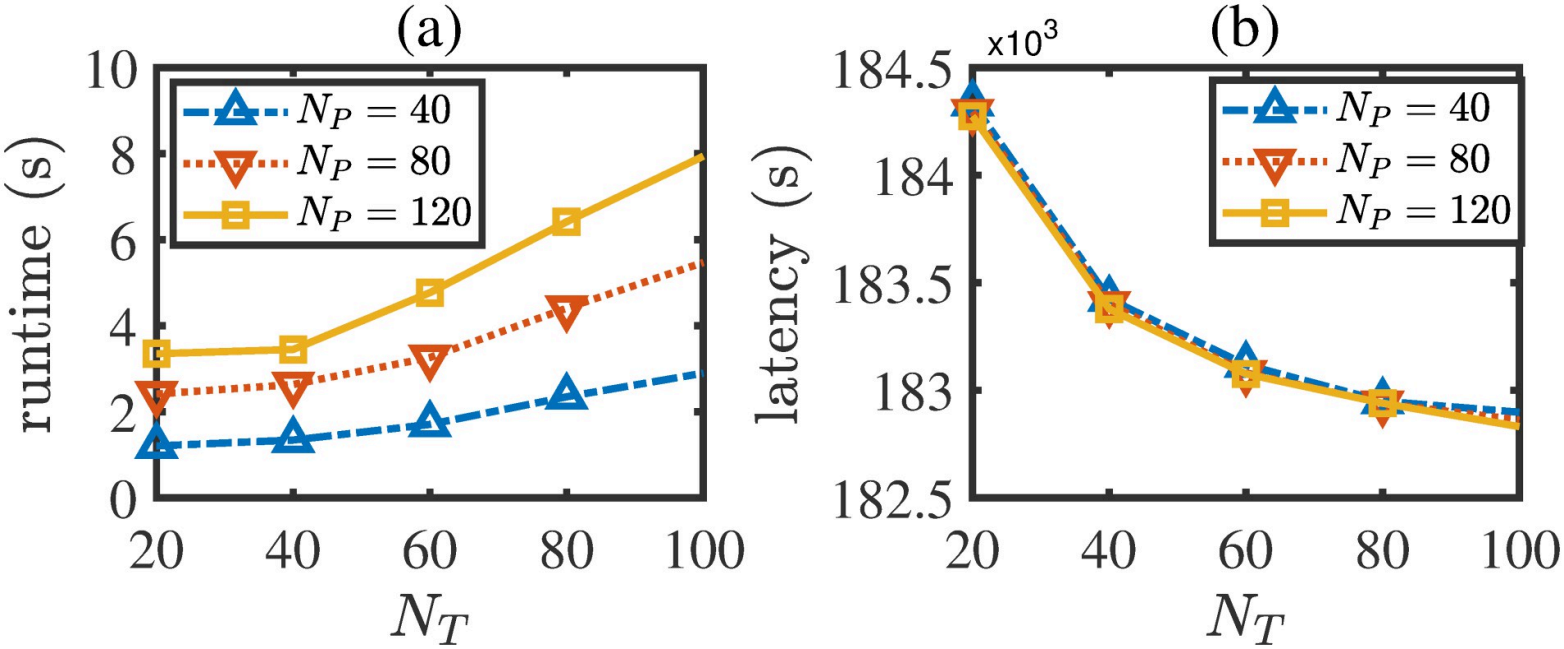
Relationship between time consumption and result quality of LBAO algorithm.

Next, let us investigate the relationship between the time consumption and result quality of the LBAO algorithm. Recall that the time complexity of the LBAO algorithm is mainly determined by the population size *N*_*P*_, the maximum iteration step *Q* and the discrete precision *N*_*T*_. Let *Q* = 100, *N*_*P*_ ∈ {40, 80, 120}, *N*_*T*_ ∈ {20, 40, 60, 80, 100}. Then, we run the LBAO algorithm for each parameter combination and record the corresponding total time consumption and task latency. The results are shown in Fig 9, from which the following two observations can be attained:

- With the increase of the discrete precision *N*_*T*_, the total time consumption of the LBAO algorithm increases in a roughly linear manner, whereas the total latency decreases slower and slower. This observation implies that it seems not a cost-efficient way to blindly increase the precision *N*_*T*_ because the resulting improvement in algorithm quality would be very limited. For example, when *N*_*P*_ = 120, if the precision *N*_*T*_ is enlarged from 20 to 100, the quality improvement would be just only $\frac{184.4-182.9}{184.4}\times 100\%\approx 0.81\%$, while the time consumption would increase by $\frac{8.0-3.5}{3.5}\times 100\%\approx 128.6\%$. On the contrary, if the precision is reduced, the algorithm time consumption will decrease quickly and the LBAO algorithm can be applied to real-time scenarios with just litter quality reduction.
- With the increase of the population size *N*_*P*_, the total time consumption of the LBAO algorithm increases dramatically while the total latency just changes slightly. This observation also suggests us not to blindly increase the population size *N*_*P*_ for better algorithm quality. Thus, we recommend setting the population size *N*_*P*_ by a small value in practical applications.
- From the above two observations and analyses, we should understand that setting the population size *N*_*P*_ and discrete precision *N*_*T*_ by small values is a cost-effective way to solve the LBAO problem. Also, from Fig 9, it is seen that when these two parameters are as small as *N*_*P*_ = 40, *N*_*T*_ = 20, the result quality is acceptable and the time consumption is just nearly one second, which is much less than the considered time horizon *T* = 6 seconds. Thus, it is reasonable to consider that the LBAO algorithm has the potential to be applied to real-time applications.

### 5.4 Influence of load balance in CEE offloading

**Fig 10:**
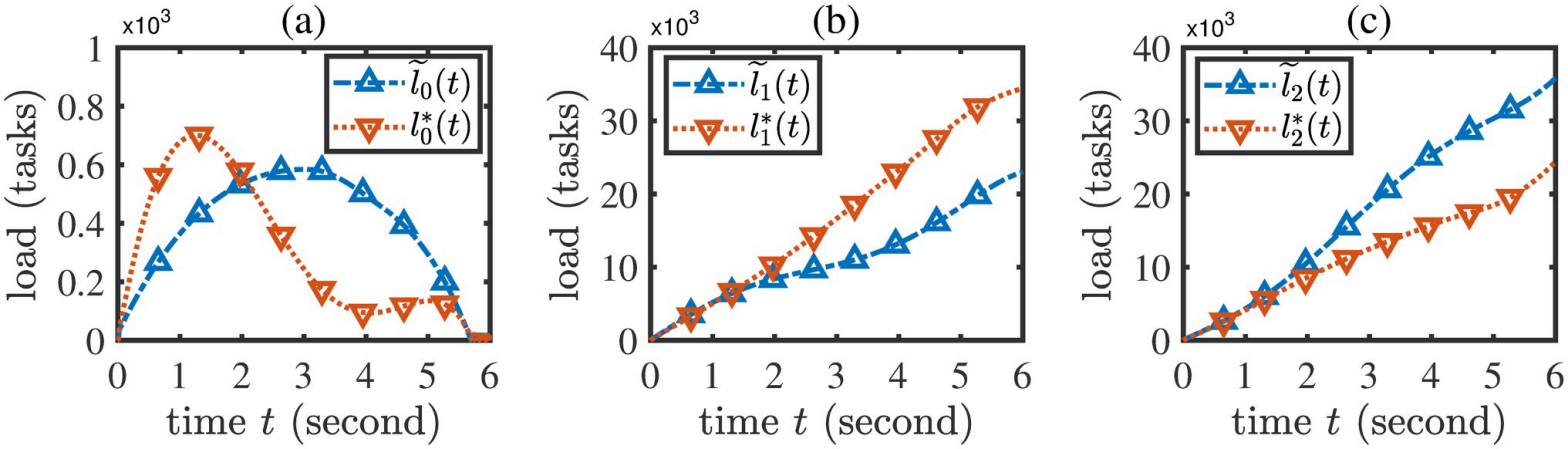
Comparison between the load evolution trajectories under the correct and erroneous optimal strategies.

Finally, let us investigate the influence of load balance in CEE collaborative offloading. To achieve that, we perform the following four experiment steps:

1. Under the actual load balance scheme *f* shown in (24), calculate the optimal strategy (x*, y*) through the LBAO algorithm and denote the corresponding load evolution trajectory by l*. The strategy (x*, y*) is the optimal decision that is aware of load balance.
2. Let the load balance scheme be *f*(*i*, *j*, *l*(*t*), *t*) ≡ 0. Calculate the optimal strategy $\left(\tilde{x},\tilde{y}\right)$ through the LBAO algorithm. This strategy means the erroneous optimal decision that ignores the effect of load balance.
3. Under the actual load balance scheme *f* shown in (24), calculate the actual load evolution trajectory $\tilde{l}$ for the erroneous strategy $\left(\tilde{x},\tilde{y}\right)$.
4. Compare the load evolution trajectories l* and $\tilde{l}$. The result is shown in Fig 10.

From Fig 10, it is seen that the influence of load balance on the optimal decision of CEE offloading strategies is non-negligible. More specifically, it follows from Fig 10A that the erroneous optimal strategy $\left(\tilde{x},\tilde{y}\right)$ tends to adopt higher cloud offloading rates because it ignores the influence of load balance and underestimates the capacity of edge servers, leading to higher load in the cloud center; on the contrary, the correct optimal strategy (x*, y*), which has accounted for the load balance scheme, tends to adopt lower cloud offloading rates because it perceives that the edge servers are able to accommodate more tasks through load balance. In addition, Fig 10B and 10C show that the load balance scheme can effectively reduce the load difference between the two edge servers. Thus, load balance is a crucial factor in determining a proper CEE offloading strategy.

## 6 Conclusion and future work

In this paper, we have addressed the LBAO problem. First, we have proposed a novel load evolution model to characterize the influences of different CEE offloading strategies on the load dynamics of a CEE system. On this basis, we have established a latency model to evaluate different CEE offloading strategies and formulated an optimal control model to describe the LBAO problem. Second, we have analyzed the feasibility of typical optimal control numerical methods in solving the LBAO problem, developed a numerical method (the LBAO algorithm) based on the GA framework to solve the LBAO problem, and made a rough analysis of the algorithm time complexity. Third, through a series of numerical experiments, we have verified the effectiveness of the LBAO algorithm.

In our research, we have shown that load balance is a crucial factor in designing CEE offloading strategies. If a strategy ignores the influence of load balance between edge servers, there would be bias in estimating the load dynamics of edge servers and the performance of offloading may not be well improved. Thus, developing a load balance -aware offloading strategy is necessary.

Still, there exist some limitations in our work. First, as we discussed earlier, it is a dilemma to simultaneously increase the result quality and decrease the time consumption of our proposed LBAO algorithm. Thus, in the future work, it would be valuable to study an improved method to address or mitigate this issue. Second, we notice that artificial intelligence (AI) -based algorithms, such as reinforcement learning [48] and adaptive dynamic programming [49], are an emerging type of numerical method to solve optimal control problems. Thus, in the future work, it is worth investigating the feasibility of AI-based methods in solving the LBAO problem. Further, if AI-based methods are available to solve the LBAO problem, it would be significant to compare their performance with our proposed GA-based algorithm. Third, in our work, the network environment in a CEE system is supposed to be relatively stable in a short time interval. In the future work, it is worth studying offloading strategies under an unstable network environment, and we may extend our work by introducing some noises in the mathematical formulation of the LBAO problem.
